# Prevalence of diabetes mellitus in a Saudi community

**DOI:** 10.4103/0256-4947.75773

**Published:** 2011

**Authors:** Khalid A. Alqurashi, Khalid S. Aljabri, Samia A. Bokhari

**Affiliations:** Division of Endocrinology, Department of Internal Medicine, King Fahad Armed Forces Hospital, Jeddah, Saudi Arabia

## Abstract

**BACKGROUND AND OBJECTIVES:**

Quantifying the prevalence of diabetes mellitus is important to allow for rational planning and allocation of resources. Therefore, we designed this study to determine the prevalence of diabetes among Saudi nationals.

**DESIGN AND SETTING:**

A cross-sectional study among patients attending a primary care clinic in June 2009.

**PATIENTS AND METHODS:**

Patients were interviewed with structured questionnaires to determine the presence of diabetes by questioning for history of the disease, and charts were reviewed to document any diabetic therapies that the patients may have undergone in the past or were undergoing at that time.

**RESULTS:**

Of 6024 subjects, diabetes mellitus was present in 1792 (30%) patients. The mean (SD) age of the patients was 55.3 (13.2) years. The prevalence of diabetes was 34.1% in males and 27.6% in females (*P*<.0001). The mean (SD) age for onset of diabetes in males and females was 57.5 (13.1) and 53.4 (13.1) years, respectively (*P*<.0001). Females <50 years old had a higher prevalence than males in the corresponding age range—34.1% and 25.1%, respectively (*P*<.0001). The prevalence of diabetes decreased in patients older than 70 years. The prevalence of body mass index of ≥25 was 72.5%. Among patients with diabetes, the prevalence of body mass index of ≥25 was 85.7% (*P*<.0001). There was a higher prevalence of obesity (body mass index, ≥25) in females (87.7%) as compared to males (83.1%) (*P*=.008).

**CONCLUSION:**

The prevalence of diabetes is high among the Saudi population and represents a major clinical and public health problem. A national prevention program to prevent diabetes and address the modifiable risk factors at the community level, targeting high-risk groups, should be implemented soon.

Diabetes mellitus is the most common chronic endocrine disorder, affecting an estimated 5% to 10% of the adult population in industrialized Western countries, Asia, Africa, Central America and South America, and it has a large impact on society.^1^–^3^ The International Diabetes Federation (IDF) estimated that there were 151 million people with diabetes in 2000. Despite methodological differences, this was similar to the present estimate for a comparable population of 147 million. The IDF has subsequently released estimates of the numbers of people with diabetes for 2003 of 194 million and forecasts for 2025 of 334 million.^4^ The clinical characteristics of the diabetic population and their comorbidities have been obtained mainly from hospitals or community-based surveys.^5^ The accompanying shift in lifestyle to more sedentary activity with higher-fat diets and resultant obesity apparently underlies much of the increased prevalence of diabetes mellitus. The Saudi population is over 18 million and is rapidly growing. Previous national health surveys have provided information on the prevalence in the northwestern, southwestern, northern, eastern and central provinces.^2^,^3^ Quantifying the prevalence of diabetes and the number of people affected by diabetes, now and in the future, is important to allow for rational planning and allocation of resources. Therefore, we describe the prevalence and characteristics of patients with diabetes mellitus as part of a cross-sectional study in the Department of Primary Care at King Fahad Armed Forces Hospital.

## METHODS

We conducted a cross-sectional study of all patients during the month of June 2009. All questionnaires were administered with an interview. Participants were asked to provide information on diabetes and medical history. Self-reported diabetes was coded as “Yes” if the participants answered having been diagnosed with diabetes, and charts were reviewed to document any diabetic therapies. Patients were selected from those attending the Department of Primary Care at King Fahad Armed Forces Hospital. The demographic data and medical history were documented. This study does not differentiate between type 1 and type 2 diabetes, as the case definition for diabetes used in the questionnaires has no sub-classifications that would allow differentiation between type 1 and type 2 diabetes. The study was approved by the ethical board of King Fahad Armed Forces Hospital.

Continuous variables were described using means and standard deviations. Univariate analyses of baseline demography and clinical laboratory endpoints, both between and within groups, were accomplished using an unpaired t test, and the chi-square test was used for categorical data comparison. Pearson correlation was used for correlation between variables and linear regression analysis used as appropriate. *P* value <.05 indicated significance. The statistical analysis was conducted with SPSS version 16.0 for Windows.

## RESULTS

**Figure 1:**
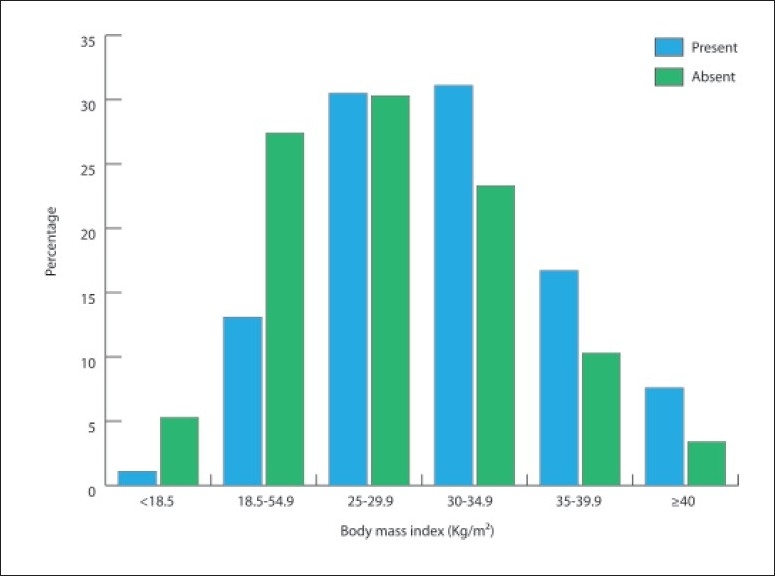
Prevalence of diabetes in groups with various ranges of body mass index.

A total of 6024 patients attending the Department of Primary Care were included in this study. There were 2279 (37.8%) males and 3744 (62.2%) females (**Table 1**). Diabetes was present in 1792 (30%) patients. Males were older than females (57.5 [13.1] vs. 53.7 [13.1] years, respectively, *P*<.0001). The prevalence of diabetes was 34.1% in males and 27.6% in females (*P*<.0001). Comparing the prevalence by gender in different age groups showed that females younger than <50 years had a higher prevalence than males in the corresponding age range, 34.1% vs. 25.1%, respectively (*P*<.0001). In males older than 50 years, the prevalence was higher when compared with females in a similar age range (**Table 2**). The prevalence of body mass index (BMI) of ≥25 was 72.5%. Among patients with diabetes, the prevalence of body mass index (BMI) of ≥25 was 85.7% (*P*<.0001), with a higher prevalence of BMI of ≥25 in females (87.7%) as compared to males (83.1%) (*P*=.008). The prevalence of diabetes in relation to BMI in the groups is shown in **Figure 1**. The distribution of BMI according to gender among patients with diabetes is shown in **Table 3**.

**Table 1:** Characteristics of patients

Parameters	Diabetes	No Diabetes
**Gender**		
Male	768 (34.1)	1486 (65.9)
Female	1024 (27.6)	2690 (72.4)
Age (years)	55.3 (13.2)	36.0 (14.8)
**Age groups (years)**		
12-19	8 (2)	400 (98)
20-29	61 (4.6)	1265 (95.4)
30-39	147 (12.1)	1070 (87.9)
40-49	326 (31.9)	696 (68.1)
50-59	558 (58.2)	401 (41.8)
60-69	447 (68.6)	205 (31.4)
≥70	245 (63.8)	139 (36.2)

**Total**	1792 (30)	4176 (70)

Data are mean (SD) or number (%).

**Table 2:** Characteristics of patients by gender and age.

Parameters	Male			Female			*P*
Age (years)	Total	Diabetes	%	Total	Diabetes	%	
12-19	180	2	1.1	228	6	2.6	.2
20-29	384	17	4.4	942	44	4.7	.4
30-39	456	65	14.3	761	82	10.8	.04
40-49	364	109	29.9	658	217	33	.2
50-59	322	194	60.2	637	364	57.1	.2
60-69	247	344	71.8	308	200	64.9	.04
≥70	204	134	65.7	180	111	61.7	.2

**Total**	2254	768	34.1	3714	1024	27.6	<.0001

Data are number (%).

**Table 3:** Characteristics of patients stratified by body mass index and gender.

Parameters	Male			Female			*P*
Body mass index (Kg/m^2^)	Total	Diabetes	%	Total	Diabetes	%	
<18.5	81	2	2.5	155	17	11.0	.02
18.5-24.9	492	120	24.4	853	101	11.8	<.0001
25-29.9	803	281	35	946	231	24.4	<.0001
30-34.9	568	222	39.1	899	300	33.4	.02
35-39.9	193	76	39.4	506	204	40.3	.4
≥40	49	21	42.9	217	106	48.8	.3

Data are number (%).

## DISCUSSION

**Figure 2:**
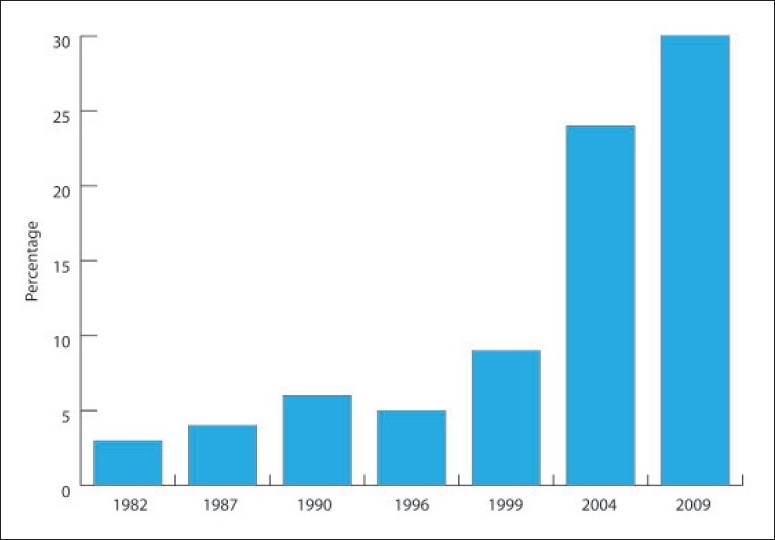
Epidemiological studies of the prevalence of diabetes in Saudi Arabia, 1982-2009.

Diabetes and obesity are multifactorial diseases of considerable heterogeneity.^6^ The prevalence of diabetes worldwide will see an increase of 42% between the years 2003 and 2025.^4^ Reported prevalence data from the Gulf region revealed high rates in Bahrain (25.7%) and Oman (16.1%).^7^,^8^ Our study showed a further increase in the prevalence of diabetes mellitus in comparison with previous studies carried out in Saudi Arabia (**Figure 2**).

Globally, diabetes prevalence is similar in males and females, but it is slightly higher in men <60 years of age and in females at older ages, which was not observed in our study. Overall, diabetes prevalence is higher in males, but there are more females than males with diabetes, which was true in our study for females in the younger age groups.^1^ The dramatic increase in the prevalence of diabetes can be explained by several factors. First, it is a disorder of the elderly. Over 20% of individuals aged 65 or more have diabetes. Studies from Saudi Arabia have shown different age-specific prevalence rates. Our data demonstrate an increasing prevalence of diabetes mellitus with advancing age; the fact that diabetes prevalence increases with age is consistent with the findings in previous studies.^1^,^2^ Second, diabetes is closely linked to obesity. The prevalence of obesity has also increased at a double-digit pace over the past decade, having risen dramatically in North America. In the United States, there has been a 61% increase in prevalence of obesity since 1991, being most likely the result of cheap, “super-sized” meals and dwindling physical activity.^9^,^10^ A higher prevalence of obesity in diabetics is well known, with 80% to 90% of people diagnosed with type 2 diabetes being obese.^11^

BMI has been proved to be a useful index for large-scale epidemiological work.^12^ The West of Scotland Coronary Prevention Study prospectively followed 5947 men for 4.9 years to predict incident of diabetes. In a multivariate regression model, BMI was found to be a significant independent predictor for diabetes.^13^ The risk of developing diabetes increases with increasing weight; and in comparison with men and women with BMI <22.5 kg/m^2^, the odds for developing diabetes increased by 119% and 116%, respectively, for those in whom the following condition was satisfied: 22.5 BMI <25 kg/m^2^.^14^ As the rate of obesity has nearly tripled in children, type 2 diabetes, virtually unheard of in adolescents a decade ago, is commonplace in pediatric clinics today. In the study of hypertensive and diabetic patients attending the primary health care clinics in Riyadh, only 19% of patients were found to have ideal weight (BMI, <25 kg/m^2^), while 35% were overweight (BMI, 25-29.9 kg/m^2^), 41% were moderately obese (BMI, 30-40 kg/m^2^) and 5% were morbidly obese (BMI >40 kg/m^2^).^15^ Another study showed that of the males studied, 20.7% were obese (BMI, 25-29.9 kg/m^2^) and 37% were overweight (BMI, >30 kg/m^2^); and among the females, 39.3% were obese and 27% were overweight.^16^ Our logistic regression results showed that BMI >25 and age, but not gender, were significantly associated with diabetes (*P*=.01, *P*≤.0001 and 0.2, respectively).

Both obesity and diabetes are preventable. Previous studies have demonstrated that changes in lifestyle are effective in preventing both diabetes and obesity in high-risk adults with impaired glucose tolerance.^17^,^18^ Increasing physical activity, improving diet and then sustaining these lifestyle changes can reduce both body weight and risk of diabetes. Health professionals must continue to stress the importance of a balanced diet and physical activity for healthy weight loss. In the Saudi society, men and women must overcome many obstacles to make the best choices for optimal health. The provision of clinical preventive services to identify and control hypertension, elevated cholesterol levels, obesity and diabetes remain important medical priorities nationally. Development and implementation of national programs to promote a balanced diet, increased physical activity and weight control must be national priorities as well.

The results of this study have three important implications for national diabetes prevention and diabetes management programs. First, it appears that diabetes prevalence rates will almost certainly continue to rise in the Saudi population over the next two decades. The rapid aging of the currently very young Saudi population to a high-risk older age, coupled with emerging life-prolonging diabetes treatments, will maintain a balance between incidence and mortality in the foreseeable future. Even if incidence rates were to flatten out or decline due to a breakthrough in diabetes prevention, prevalence rates would continue to rise as incidence outpaces mortality. As a result, the health burden due to all types of diabetic complications will likely continue. This means that the health care and social service systems should start preparing now to provide the secondary prevention and support services and systems that a large number of adults with diabetes are going to require to maintain a reasonably good quality of life. These include diabetes-screening programs, foot-care programs, accessible dialysis services, dietary counseling services and an enhanced infrastructure at the community level to facilitate independent living by adults with limited mobility and eyesight. Second, “upstream” population-based primary prevention programs need to be aggressively implemented to ensure that diabetes incidence begins to decrease in the future. The dramatically higher rates of diabetes in the Saudi population highlight the urgency of this activity. Because diabetes appears to be closely related to the adoption of many aspects of the modern lifestyle, including diet and low levels of physical activity, prevention programs that draw upon traditional practices need to be implemented. A number of very promising primary prevention programs that draw upon older traditions and ways of life have been implemented in our institution. Third, the reason for the higher prevalence of diabetes in Saudi women below 50 years of age observed in this study also needs to be better understood. The relationship to earlier episodes of gestational diabetes should be investigated as one possibility.

This study has methodological limitations that must be kept in mind when interpreting its results. National health surveys depend commonly on data collected through self-administered questionnaires due its lower costs. In several studies, self-reported data were compared with data from medical records, disease registries or the results of clinical and laboratory investigations. Our data has relied exclusively on data derived from a self-reported questionnaire, along with interview with patients to estimate diabetes prevalence. Since this approach depends upon diabetes cases being recognized, diagnosed and recorded. Self-reporting information of diabetes can lead to inaccurate estimates of its prevalence rates; however, self-reporting of diabetes has a better validity than that of other chronic diseases.^19^ This study does not differentiate between type 1 and type 2 diabetes, but given that type 2 diabetes constitutes 90% to 95% of all diabetes cases, the use of ‘diabetes’ in this study is likely a valid proxy for ‘type 2 diabetes’.

To conclude, this study has provided epidemiological information on the extent of diabetes mellitus as a health problem and has emphasized the value of having accurate population-based information on the epidemiology of diabetes in our population for future planning and implementation. By providing information on the trend and the geography of diabetes in our center, this study provides important clues as to the magnitude and structure of the primary and secondary intervention programs that will be required to effectively manage this disease.
